# A roadmap for a comprehensive diagnostic approach to fine needle cytology of lymph node metastases

**DOI:** 10.1111/cyt.13172

**Published:** 2022-09-05

**Authors:** Gennaro Acanfora, Antonino Iaccarino, Filippo Dello Iacovo, Pasquale Pisapia, Caterina De Luca, Claudia Giordano, Claudio Bellevicine, Marco Picardi, Giancarlo Troncone, Elena Vigliar

**Affiliations:** ^1^ Department of Public Health University of Naples "Federico II" Naples Italy; ^2^ Hematology Section, Department of Clinical Medicine and Surgery University of Naples "Federico II" Naples Italy

**Keywords:** cell block, fine needle cytology, lymph node, metastases

## Abstract

**Objective:**

Fine needle cytology (FNC) is widely used as a first‐line procedure in the diagnostic algorithm of lymphadenopathies. In a metastatic setting, a first‐line diagnostic approach identifies non‐haematopoietic malignancy; however, cytopathologists could also provide a second diagnostic level, identifying the origin of the primary tumour. This paper outlines a comprehensive and practical approach to the cytological diagnosis of lymph node metastases.

**Methods:**

Cytological diagnoses of lymph node metastases performed over a 10‐year period were selected and divided into two groups. The first group, labelled “oncological,” comprised patients with a previous history of malignancy; the second group, labelled “naïve,” included patients with no relevant history. Pathology records were retrieved to record microscopic findings, namely, background appearance, group architecture, and specific cell features; data from cell block (CB) preparations were also collected.

**Results:**

Overall, 982 cases were selected: 497 cases (50.61%) in the naïve group, and 485 (49.39%) in the oncological group. Overall, a second diagnostic level was achieved in 834/982 cases (84.92%); cases diagnosed as carcinoma not otherwise specified were more frequent in the naïve group than in the oncological group (17.51% vs. 8.04%, *P* < 0.01). Notably, although CB material was available in only 44.87% of the naïve cases, we were able to achieve a second diagnostic level thanks to the integration of clinical and cytomorphological findings, plus lymph node topography, in 82.49% of the cases.

**Conclusion:**

Our results confirmed that in a metastatic setting, FNC can reliably lead to the identification of the origin of the primary tumour.

## Graphical abstract

Schematic correlation between lymph node localisation, distribution of the final diagnoses, and the corresponding relevant cytological features observed in a series of 982 lymph node metastases fine needle cytology samples

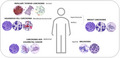

## 1 INTRODUCTION

Fine needle cytology (FNC) is widely used as a first‐line procedure in the diagnostic algorithm of lymph node enlargement. In fact, although excisional biopsy and histopathological examination remain the gold standard,^1^ FNC can reliably diagnose benign, reactive, or infective lymphadenopathies, thereby avoiding unnecessary surgery.^2^, ^3^, ^4^ Moreover, histological confirmation of cytological findings is not mandatory in several cases including Hodgkin and non‐Hodgkin lymphoma relapses, metastases from known primary tumours, primary lymphomas, and metastases of unknown primary origin in patients in which surgery is either contraindicated or impossible.^5^

In a metastatic setting, a first diagnostic level allows cytopathologists to identify non‐haematopoietic malignancies. However, according to the recent classification and reporting system of lymph node FNC, in addition to the first diagnostic level, cytopathologists could also provide a second diagnostic level, matching clinical and morphological features with ancillary test results.^5^

Knowing the clinical presentation and, in particular, the topographic site of lymphadenopathies, is critical to identifying primary neoplasms. In this regard, tumour‐draining lymphatic vessel pathways may provide crucial information about early metastases.^6^ Generally, upper cervical lymph nodes are involved in neoplasms of the head and neck district, whereas lower cervical lymph nodes are involved in thoracic or abdominal tumours; axillary metastases most frequently originate from breast cancer in women and from lung cancer in men; abdominal and pelvic/inguinal lymph node metastases usually originate in the neoplasms of reproductive and non‐reproductive abdominal and pelvic organs.^7^

Non‐invasive cytological examination can provide additional diagnostic information about the nature and type of lymph node lesions. For instance, cytological findings indicating a different proportion of lymphoid and metastatic cells could be the expression of a partial or complete lymph node replacement. Moreover, FNC can also identify some specific morphological features, indicative of certain types of tumour differentiation, such as adenocarcinomas and sarcomas, as well as a pool of possible primary origins. Although the cytological features of metastases from almost any type of malignant tumour have been described, most of the published data generally focus on FNC of metastatic lymph nodes from single specific neoplasms or from single topographic locations.^8^, ^9^, ^10^, ^11^, ^12^, ^13^, ^14^, ^15^, ^16^, ^17^ Unfortunately, the reality in routine clinical practice is much more complex than that. Indeed, lymphadenopathies of various anatomical districts still pose a great diagnostic challenge for cytopathologists. Therefore, in this paper, we outline a comprehensive and practical approach to the cytological diagnosis of lymph node metastases on the basis of a 10‐year experience in running a referral FNC clinic.

## 2 MATERIALS AND METHODS

The electronic database of the Cytopathology Division at the University of Naples Federico II was searched to obtain the diagnostic reports of lymph node FNCs performed over a 10‐year period, specifically between January 2009 and December 2019; cases with a final cytological diagnosis of metastasis were selected. In all cases, FNCs were performed under ultrasound (US) guidance by an experienced cytopathologist. In cases of deeply located lymph nodes, FNCs were assisted by a haematologist with more than 15 years of experience with interventionist power‐doppler US; no endoscopic/endobronchial US‐guided transbronchial needle aspiration was performed. A 23‐gauge needle was used. The first pass served to prepare a direct smear, which was stained with Diff‐Quik (Bio Optica S.p.A) for rapid on‐site evaluation and microscopically evaluated for adequacy assessment and specimen triage. Residual material in the needle and from additional passes was smeared and immediately alcohol‐fixed for Papanicolaou staining and/or collected and suspended in 5 mL of 10% neutral buffered formalin for cell block (CB) preparation in case immunocytochemistry (ICC) was deemed necessary.

Pathology records were retrieved, and patients' clinical and pathological information was recorded, namely, age, sex, lymph node location, clinical history, and final diagnosis. In addition, the data on CB preparation, and number and types of ICC staining performed were also recorded.

Data were globally analysed by splitting the case series into two groups: (1) an “oncological group,” comprising patients with a previous history of malignancy; and (2) a “naïve group,” involving patients with lymphadenopathies of unknown origin. Global differences between the two groups were assessed with the chi‐squared test; *P* values < 0.05 were deemed to be statistically significant. Moreover, in the naïve group, each entry was matched with a histological follow‐up, if available, to evaluate the cytohistological concordance in terms first and second diagnostic levels; in particular, cases diagnosed as sarcoma or carcinoma not otherwise specified (NOS) were considered as discordant. All information regarding human material was managed using anonymous numerical codes, and all samples were handled in compliance with the Declaration of Helsinki.

### 2.1 Cytomorphological features

**FIGURE 1:**
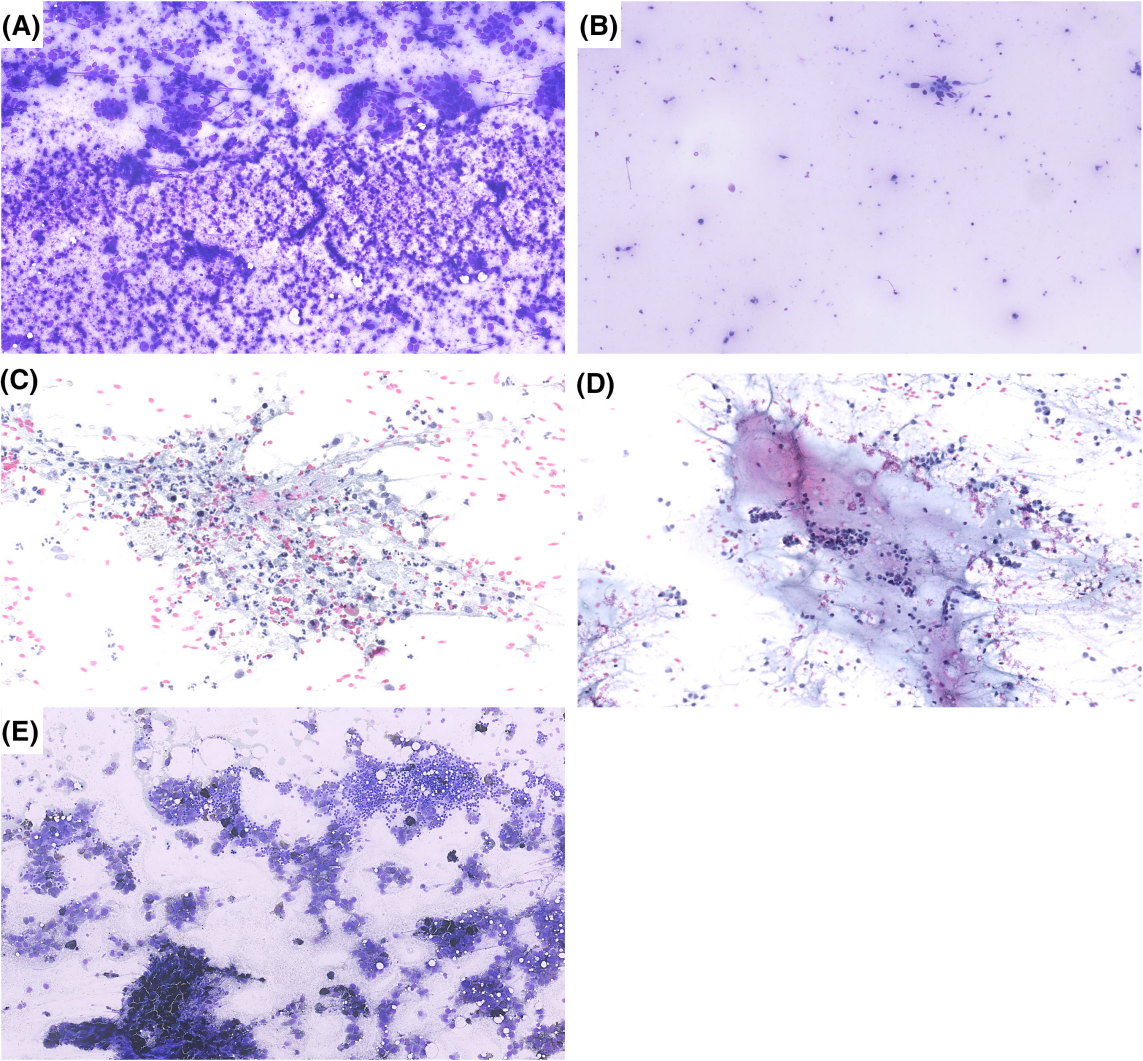
Relevant microscopic findings in relation to background appearance. (A) Necrosis. (B) Fluid. (C) Inflammation. (D) Mucus. (E) Pigment. (A‐C, E: Diff‐Quik, 10×; C, D: Papanicolaou, 10×)

**FIGURE 2:**
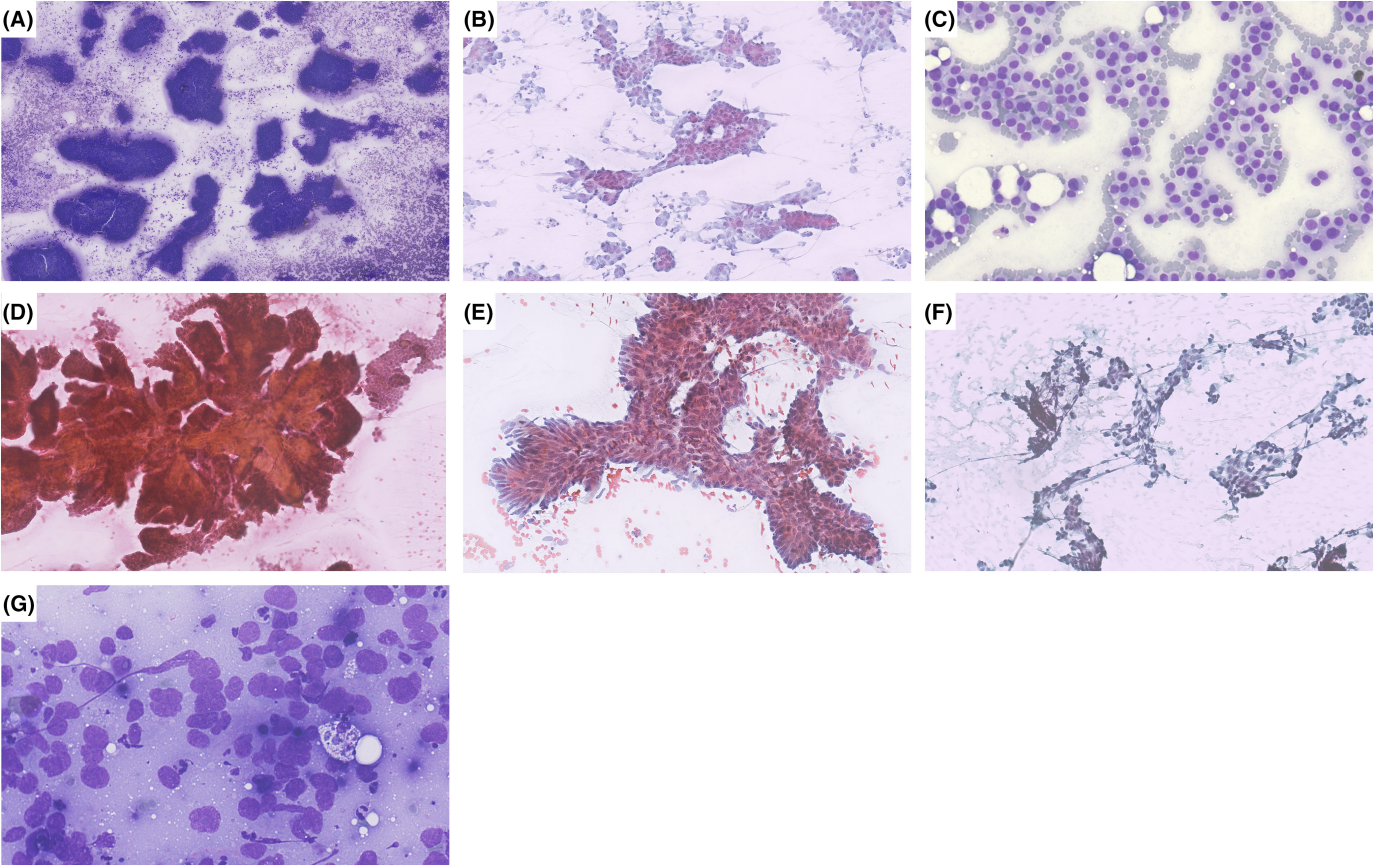
Relevant microscopic findings in relation to group architecture. (A) Solid. (B) Glandular. (C) Dyscohesive cells. (D) Papillae. (E) “Picket fence.” (F) Crushing artefact. (G) Moulding. (A: Diff‐Quik, 5×; B, D‐F: Papanicolaou, 20×; C, G: Diff‐Quik, 20×)

**FIGURE 3:**
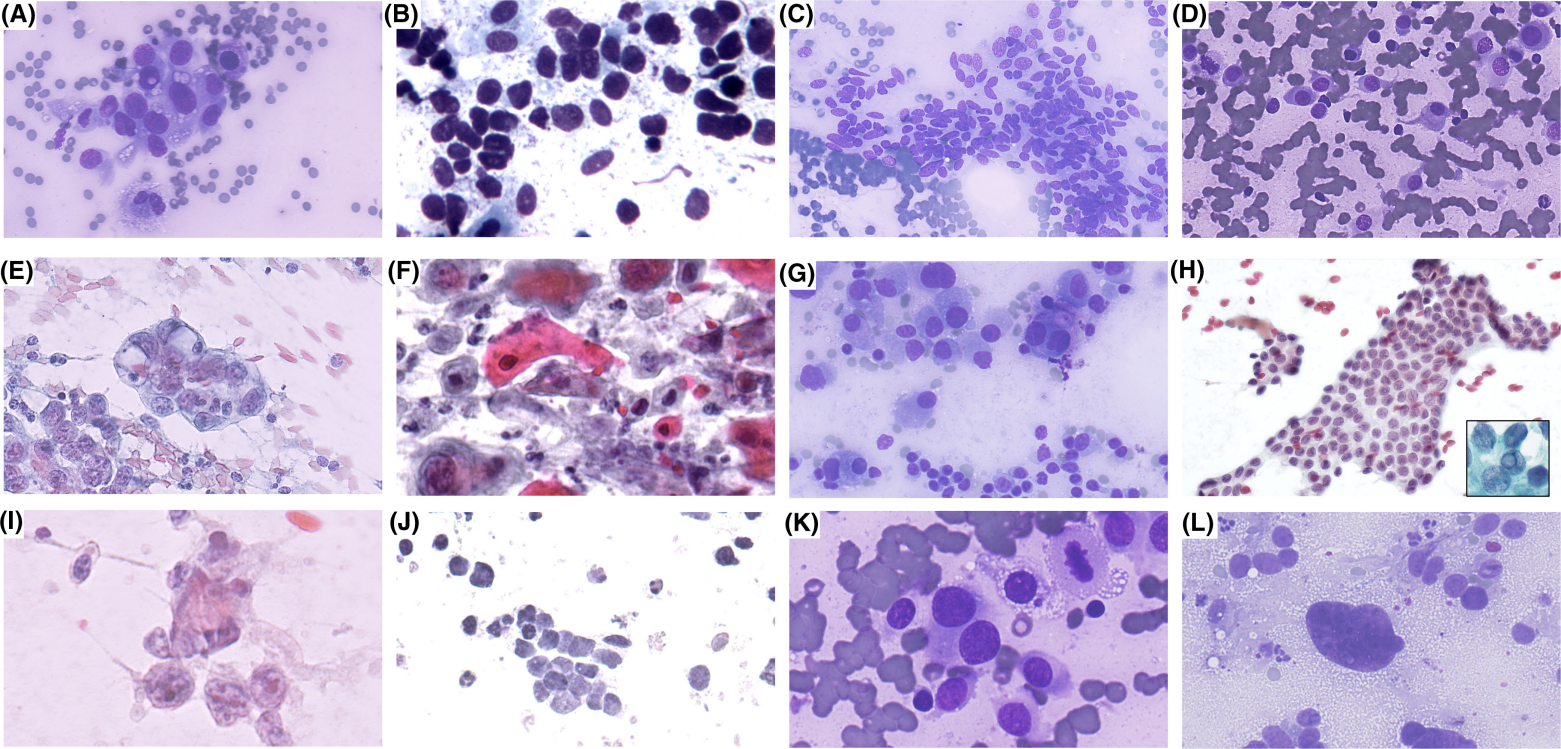
Relevant microscopic findings in relation to specific cytological features. (A) Large cells. (B) Small cells. (C) Spindle‐fusiform cells. (D) Plasmacytoid cells. (E) Cytoplasm vacuolisation. (F) Squamoid appearance. (G) Granular cytoplasm. (H) Chromatin clearing and grooves; intranuclear cytoplasmic inclusion in the inset. (I) Prominent nucleoli. (J) Granular chromatin. (K) Binucleation. (L) Anaplasia (A, C, D, G, K, L: Diff‐Quik stain, 40×; B, E, F, H, I, J: Papanicolaou; 40×)

Pathology records were also retrieved to record relevant microscopic findings associated with background appearance, group architecture, and specific cell features. In particular, the background appearance included the presence of necrosis, fluid, inflammation, mucus, pigment, and calcification. Group architecture was evaluated rather according to the following categories: solid, glandular, papillary, dyscohesive cells, “picket fence”, crushing artefact, and moulding. Finally, cytological features predictive of malignancy were the presence of atypical cell dimension and shape (large cells, spindle‐fusiform cells, plasmacytoid cells), cytoplasm dimension and appearance (large cytoplasm, scant cytoplasm, vacuolisation, squamoid appearance, and granular cytoplasm), and nuclear atypia (intranuclear cytoplasmic inclusion [INCI], grooves, prominent nucleoli, granular chromatin, chromatin clearing, binucleation, and anaplasia) (Figures 1, 2, 3). Lastly, the final cytological diagnoses were correlated with the above‐mentioned findings and the location of the lymphadenopathies.

## 3 RESULTS

Between 1 January 2009 and 31 December 2019, our cytopathology laboratory performed 4259 lymph node FNCs. This 10‐year period was chosen to ensure a clinical follow‐up of at least 24 months. In brief, we selected *n* = 982/4259 cases (23%) diagnosed as metastases. US‐guided FNCs were performed on patients of all ages, ranging from 13 to 93 years (mean age 58 years). Of these, 614 patients were females (62.53%), and 368 were males (37.47%). The anatomical sites of the lymph nodes were the following: cervical (*n* = 329, 33.5%), axillary (*n* = 319, 32.48%), superclavicular (*n* = 178, 18.13%), inguinal (*n* = 60, 6.11%) mandibular/preauricular (*n* = 54, 5.5%), abdominal (*n* = 27, 2.75%), and thoracic (*n* = 15, 1.53%).

FNCs were performed on 497 cases (50.61%) with no history of malignancy (naïve group) and on 485 cases (49.39%) with a previous oncological history (oncological group). In particular, in 181 patients (18.43%), FNC was concurrently performed on the suspected primary lesion and the lymphadenopathy. In *n* = 589 cases (59.98%) a CB was not prepared, whereas in *n* = 393 instances (40.02%) additional material for CB preparation was collected during the FNC procedure; of these CBs, *n* = 22/393 (5.59%) were deemed non‐contributory, owing to scant or absent cellularity. Basic clinical data and features of the 982 FNCs performed on metastases are summarised in Table 1.

**TABLE 1:** Clinical data and sample features of 982 consecutive cases of lymph node metastases diagnosed on fine needle cytology between 1 January 2009 and 31 December 2019

	*n*	%
Sex		
Female	614	62.53
Male	368	37.47
Age		
Minimum	13	
Maximum	93	
Mean	5814	
Medical history		
Previous pathological diagnosis	485	49.39
No relevant history	497	50.61
Lymph node location		
Cervical group	329	33.50
Axillary group	319	32.48
Supraclavicular	178	18.13
Inguinal	60	6.11
Mandibular/preauricular	54	5.5
Abdominal	27	2.75
Thoracic	15	1.53
Cell Block preparation		
No	589	59.98
Yes	393	40.02

**FIGURE 4:**
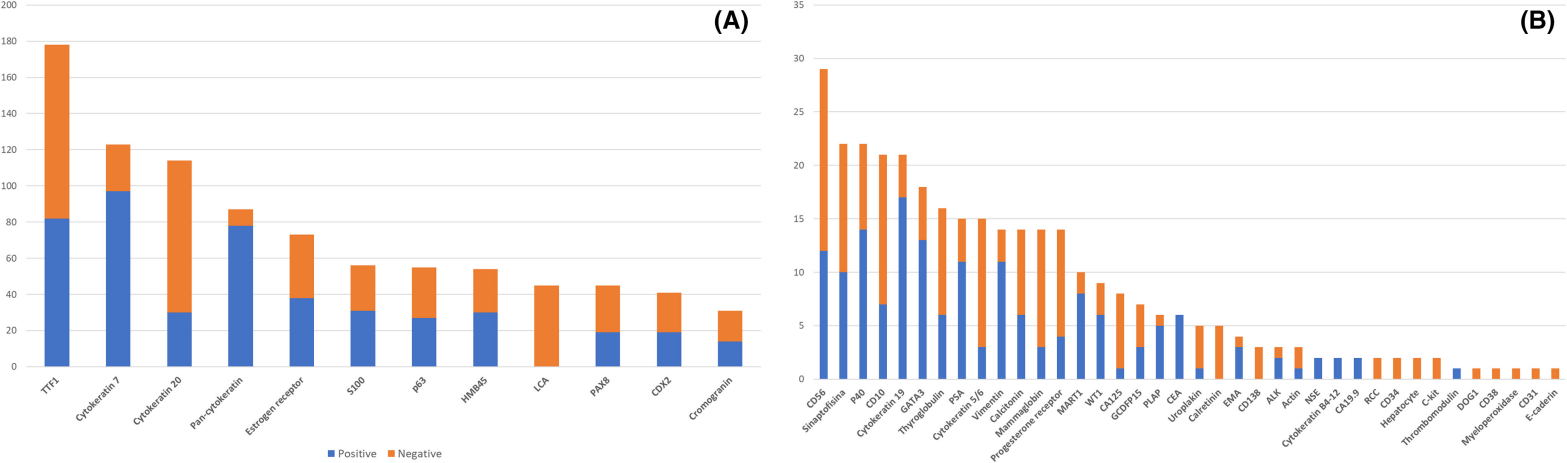
Type and positivity proportion of immunocytochemistry (ICC) staining. (A) ICC performed in more than 30 cases. (B) ICC performed in fewer than 30 cases

CBs were prepared from 170/485 cases in the oncological group and from 223/497 cases in the naïve group (35.05% vs. 44.87%; *P* < 0.01). Overall, ICC staining was performed in *n* = 371/982 cases (37.7%); in terms of just the number of cases in which a CB was prepared, ICC was performed in 371/393 CBs (94.4%). In particular, the number of ICC stains per case ranged from 1 to 15 with a median number of 3.86 immunostainings per sample. The types and proportions of ICC‐positive cases are reported in Figure 4.

As for the final cytological diagnoses, sarcoma and carcinoma NOS were diagnosed in 4 (0.41%) and 126 cases (12.83%), respectively. In detail, carcinoma NOS was diagnosed in 39/485 cases in the oncological group and in 87/497 cases in the naïve group (8.04% vs. 17.51%; *P* < 0.01) (Table 2). Conversely, in 834/982 cases (84.92%), the specific primary diagnosis was identified or suggested, and no statistically significant differences in content were observed between the two groups, apart from papillary thyroid carcinoma, melanoma, colorectal colon, and ovarian cancer, which were more frequent in the oncological group (Table 2).

**TABLE 2:** Distribution of primary tumours in lymph node metastases in the overall series and in the oncological and naïve groups

Primary neoplasms	Total *n*	%	Oncological group *n*	%	Naïve group *n*	%	*p‐value*
Breast carcinoma	308	31.36	141	29.07	167	33.60	>0.05
Squamous cell carcinoma	153	15.58	70	14.43	83	16.70	>0.05
Papillary thyroid carcinoma	144	14.66	86	17.73	58	11.67	**<0.01**
Non‐small cell lung cancer	73	7.43	34	7.01	39	7.85	>0.05
Melanoma	56	5.70	39	8.04	17	3.42	**<0.01**
Colorectal cancer	20	2.04	17	3.51	3	0.60	**<0.01**
Small cell lung cancer	14	1.43	4	0.82	10	2.01	>0.05
Ovarian cancer	13	1.32	10	2.06	3	0.60	**<0.05**
Medullary thyroid carcinoma	11	1.12	7	1.44	4	0.80	>0.05
Prostatic cancer	9	0.92	3	0.62	6	1.21	>0.05
Urothelial carcinoma	8	0.81	6	1.24	2	0.40	>0.05
Renal cell carcinoma	6	0.61	3	0.62	3	0.60	>0.05
Gastric cancer	6	0.61	4	0.82	2	0.40	>0.05
Endometrial cancer	6	0.61	5	1.03	1	0.20	>0.05
Neuroendocrine carcinoma	5	0.51	3	0.62	2	0.40	>0.05
Merkel cell carcinoma	5	0.51	4	0.82	1	0.20	>0.05
Follicular thyroid carcinoma	5	0.51	3	0.62	2	0.40	>0.05
Seminoma	4	0.41	3	0.62	1	0.20	>0.05
Pancreatic ductal carcinoma	2	0.20	1	0.21	1	0.20	ns
Anaplastic thyroid cancer	2	0.20	1	0.21	1	0.20	ns
Cholangiocarcinoma	2	0.20	1	0.21	1	0.20	ns
Not reached. carcinoma NOS	126	12.83	39	8.04	87	17.51	**<0.01**
Not reached, sarcoma NOS	4	0.41	1	0.2	3	0.6	ns
	982		485		497		

*Note*: Statistically significant *p*‐value in bold

Abbreviations: ns, insufficient sample size; NOS, not otherwise specified.

Concerning the naïve group, histopathological correlation was available in 212/497 cases (42.65%); the remaining cases were treated on the basis of the cytological results or were lost to follow‐up. In this first diagnostic level, the identification of a non‐haematopoietic malignancy was confirmed in 212/212 cases (100%). Moreover, in the cytological second diagnostic level, the identification of the primary origin of the tumour was confirmed in 180/212 cases (84.9%). Of these, 50 cases had CB material available for ICC staining, whereas 130 cases were diagnosed on the basis of cytomorphological features on direct smears and clinical presentation. In particular, the most frequent diagnosis was represented by metastasis from breast carcinoma (*n* = 100), followed by papillary thyroid carcinoma (*n* = 15), squamous cell carcinoma (*n* = 11), gastric cancer (*n* = 2), melanoma (*n* = 1), and medullary thyroid carcinoma (*n* = 1). Out of the 32 discordant cases, 31 were cytologically diagnosed as carcinoma NOS; in 1 case, in which ICC positivity for cytokeratin‐7 and negativity for oestrogen receptors suggested a cytological diagnosis of metastasis from lung, a histological control revealed a metastasis from breast carcinoma. Interestingly, unlike the results from the ICC analysis, the histological material revealed a focal positivity for oestrogen receptors.

### 3.1 Cytomorphological features

Overall, the presence of a specific and non‐haemorrhagic background was described in *n* = 238 cases (24.2%); one or more types of group architecture were described in *n* = 816 (83.09%) cases. Finally, one or more specific cytological features were described in *n* = 535 reports (54.4%); in the remaining cases (45.6%), non‐otherwise specified “cytological atypia” were reported.

In particular, in cases in which a non‐haemorrhagic background type was described, necrosis was frequently observed in several neoplasms (small cell lung cancer [SCLC; 100%], ovarian serous cell carcinoma [80%], lung adenocarcinoma [64.29%], squamous cell carcinoma [61.84%], and colon carcinoma [60%]); mucoid background was also frequently described in metastases from colon carcinoma (40%). Fluid background was frequently observed in metastases from papillary thyroid carcinoma (83.33%); conversely, lower percentages of other background types (up to 30%) were observed.

Regarding group architecture, a solid pattern was the most frequently described in several metastases (squamous cell carcinoma [83.06%], breast carcinoma [81.95%], lung adenocarcinoma [67.14%]). Dyscohesive cells were also observed in few other cancers (medullary thyroid carcinoma [100%], SCLC [100%], and melanoma [92.16%]). Conversely, papillary and picket fence appearance was mainly described in specific diagnoses (ovarian and papillary thyroid carcinoma [75% and 72.95%, respectively], and colon carcinoma [33.33%]). Likewise, high percentages of specific cytological features were described in specific entities. Among these were plasmacytoid appearance in medullary thyroid carcinoma (89.89%) and melanoma (50%); scant cytoplasm in SCLCs (53.85%), vacuolisation in colon, breast, and lung adenocarcinoma (75%, 50.57%, and 48%, respectively); squamoid appearance in squamous cell carcinoma (89.43%); and INCIs and grooves in papillary thyroid carcinoma (77.98% and 69.72%, respectively). Data are summarised in Table 3.

**TABLE 3:** Distribution of cytomorphological features described in lymph node metastases; primary origin diagnosed at least in 10 cases were considered

	Breast carcinoma	Papillary Thyroid Carcinoma	Carcinoma NOS	Squamous cell carcinoma	Non‐small cell lung cancer	Melanoma	Colon carcinoma	Ovarian carcinoma	Medullary thyroid carcinoma	Small cell lung cancer	Total
Background	31	42	41	76	14	16	10	5	‐	3	238
Necrotic	45.16% (14)	‐	41.46% (17)	61.84% (47)	64.29% (9)	18.75% (3)	60.00% (6)	80.00% (4)	‐	100% (3)	
Fluid	25.81% (8)	83.33% (35)	17.07% (7)	15.79% (12)	‐	18.75% (3)	‐	‐	‐	‐	
Proteinaceous	22.58% (7)	‐	9.76% (4)	‐	%14.29 (2)	31.25% (5)	‐	‐	‐	‐	
Inflammatory	‐	7.14% (2)	31.71% (13)	22.37% (17)	7.14% (1)	‐	‐	‐	‐	‐	
Mucoid	6.45% (2)	‐	‐	‐	‐	‐	40.00% (4)	‐	‐	‐	
Pigmentation	‐	‐	‐	‐	‐	31.25% (5)	‐	‐	‐	‐	
Calcification	‐	9.52% (4)	‐	‐	‐	‐	‐	20% (1)	‐	‐	
Group architecture	277	122	122	124	70	51	18	12	9	11	816
Solid	81.95% (227)	27.87% (34)	58.20% (71)	83.06% (103)	67.14% (47)	17.65% (9)	44.44% (8)	41.67% (5)	22.22% (2)	41.67% (5)	
Glandular	1.44% (4)	‐	5.60% (7)	‐	15.71% (11)	‐	38.89% (7)	‐	‐	‐	
Dyscohesive cells	39.71% (110)	‐	35.25% (43)	45.97% (57)	48.57% (34)	92.16% (47)	‐	16.67% (2)	100% (9)	91.67% (11)	
Papillary	2.53% (7)	72.95% (89)	2.46% (3)	‐	10.00% (7)	‐	‐	75.00% (9)	‐	‐	
"Picked fence"	‐	‐	‐	‐	‐	‐	33.33% (6)	‐	‐	‐	
Crushing	0.36% (1)	‐	3.28% (4)	‐	‐	‐	‐	‐	‐	16.67% (2)	
Moulding	‐	4.10% (5)	6.56% (8)	‐	1.43% (1)	‐	‐	‐	11.11% (1)	75.00% (9)	
Cytological features	87	109	73	123	50	52	8	11	9	13	535
Cells dimension and shape											
Large	‐	‐	18.84% (13)	10.57% (13)	36.00% (18)	40.38% (21)	‐	‐	11.11% (1)	‐	
Small	11.49% (10)	‐	13.04% (9)	0.81% (1)	‐	‐	‐	‐	‐	90.91% (10)	
Spindle‐fusiform	‐	0.92% (1)	‐	‐	‐	13.46% (7)	27.50% (3)	‐	11.11% (1)	‐	
Plasmacytoid	32.18% (28)	2.75% (3)	15.94% (11)	‐	8.00% (4)	50.00% (26)	‐	‐	88.89% (8)	‐	
Cytoplasm dimension and appearance											
Large	19.54% (17)	3.67% (4)	2.90% (2)	‐	12.00% (6)	3.85% (2)	‐	‐	‐	‐	
Scant	‐\	‐	‐	2.44% (3)	‐	‐	‐	27.27% (3)	‐	53.85% (7)	
Vacuolisation	50.57% (44)	‐	28.99% (20)	‐	48.00% (24)	7.69% (4)	75.00% (6)	‐	‐	‐	
Squamoid	‐	16.51% (18)	7.25% (5)	89.43% (110)	‐	1.92% (1)	‐	‐	‐	‐	
Granular	‐\	‐	‐	‐	‐	‐	‐	18.18% (2)	11.11% (1)	‐	
Nuclear atypia											
INCI	‐	77.98% (85)	2.90% (2)	‐	10.00% (5)	15% (28.85)	‐	‐	11.11% (1)	‐	
Grooves	‐	69.72% (76)	‐	‐	‐	3.85% (2)	‐	‐	‐	‐	
Prominent nucleoli	17.24% (15)	1.83% (2)	8.70% (6)	0.81% (1)	28.00% (14)	32.69% (17)	‐	36.36% (4)	11.11% (1)	‐	
Granular chromatin	‐	‐	5.80% (4)	‐	‐	‐	‐	‐	‐	38.46% (5)	
Chromatin clearing	‐	11.01% (12)	‐	‐	16.00% (8)	‐	‐	‐	44.44% (4)	‐	
Binucleation	11.49% (10)	‐	10.14% (7)	‐	‐	55.77% (29)	‐	18.18% (2)	‐	‐	
Anaplasia	2.30% (2)	‐	8.70% (6)	‐	‐	‐	‐	‐	‐	‐	

*Note*: Overall, the presence of specific, non‐haemorrhagic background, was described in *n* = 238 cases, the identification of one or more groups architecture was reported in *n* = 816 cases and one or more specific cytological features were described in *n* = 535 reports. Percentages refers to the total of cases in which a specific cytomorphological feature is described.

Abbreviations: INCI, intranuclear cytoplasmic inclusion; NOS, not otherwise specified.

**FIGURE 5:**
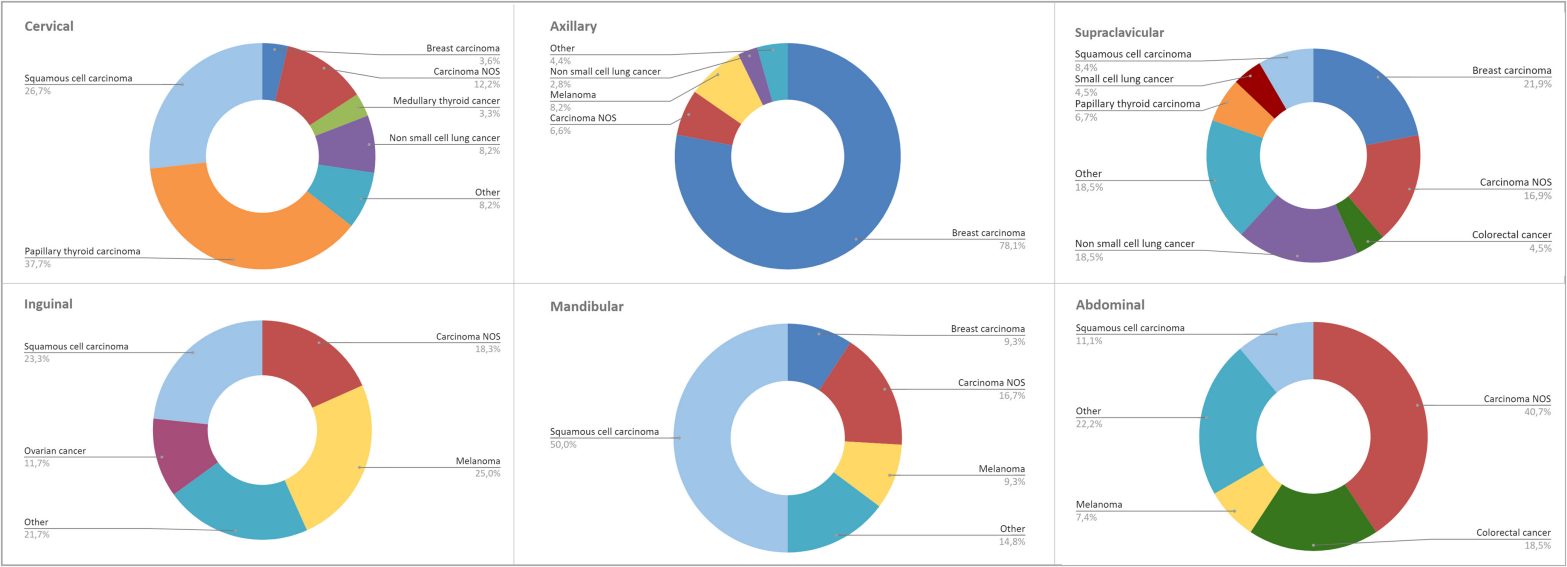
Distribution of primary origin of lymph node metastases with regard to the lymph node localisation

Regarding correlation between the identification of the site of primary origin and the localisation of lymph nodes, our data showed that cervical papillary thyroid carcinoma (37.69%) and squamous cell carcinoma (26.75%) were the most frequently diagnosed cancers in the cervical lymph node group, and breast cancer was the most frequently diagnosed cancer in the axillary lymph node group (78.06%) and in supraclavicular lymph nodes (21.91%), followed by lung adenocarcinoma (18.54%). Melanoma and squamous cell carcinoma (25% and 23.33%, respectively) were most frequently diagnosed in the inguinal group. Squamous cell carcinoma (50%) and carcinoma NOS (16.67%) were most frequently diagnosed in the mandibular/preauricular lymph nodes. Also, carcinoma NOS (57.89%) and colon‐rectal carcinoma (26.32%) were the most common primary neoplasms in the abdominal group. Finally, papillary thyroid carcinoma (71.43%) and carcinoma NOS (57.14%) were most frequently diagnosed in the thoracic lymph node group (Figure 5).

## 4 DISCUSSION

The identification of non‐haematopoietic malignancies in lymph nodes is generally quite straightforward. However, the diagnostic accuracy of suspicious cases can be improved by considering the histotypes of known primary tumours and the topography of lymph nodes; moreover, in cases of unknown primary origin, peculiar cytomorphological findings can reveal diagnostically relevant information.^7^ In our series, although specific primary diagnoses were identified or suggested in most cases (84.92%), the number of carcinoma NOS cases was higher in the naïve group (21.13%) than in the oncological group (8.04%; *P* < 0.01), confirming the relevant role of clinical history in achieving diagnostic accuracy. Equally important for the diagnosis of suspicious metastases is the topography of lymph nodes. Indeed in the present series, the distribution of cancer types in different node groups confirmed predictions based on lymphatic drainage pathways. However, cytopathologists should also take into account that such distribution may be influenced by additional factors, including geographical issues, like variability in the incidence rates of tumours in different countries.^10^ Moreover, the availability of specialised cancer facilities and the intra‐institutional range of specialties and services also affect diagnosis distribution. For example, in our series, although the most frequent primary diagnosis in the axillary group was represented by breast carcinoma, the observed percentage was remarkably higher (78%) than that of other frequently expected tumours such as lung cancer (non‐small cell lung cancer [NSCLC], 2.82%, and SCLC, 0.63%). We speculate that this phenomenon was attributable to the presence of an institutional multidisciplinary breast clinic, which led to the prevalence of breast cancer patients in our FNC clinic,^18^ and, conversely, to the referral of NSCLC patients to nearby hospitals for staging. This point is also supported by the low number of thoracic lymph nodes evaluated in our series.

Our findings also highlight the relevance of a detailed cytomorphological evaluation of cytological material from suspicious metastatic lymph nodes. In particular, the entire cytological context^5^, ^7^ in terms of background, group architecture, and cytological atypia may greatly help to identify or suggest the specific primary origin of tumours, regardless of the ICC findings. For example, we found that the main features of metastases from papillary thyroid carcinoma were a fluid background (83.33%), indicative of the frequent cystic changes observed in lymph node metastases,^19^ along with papillary architecture (72.95%), INCIs (77.98%), and grooves (69.72%). Further, the presence of necrotic (60%) or mucoid (40%) background coupled with solid, glandular, or picket fence architecture was a hallmark of metastases from colon carcinoma. Similarly, the presence of a dyscohesive cell population (92.16%), frequently binucleated (55.77%), in a pigmentated background (31.25%) was indicative of melanoma. Conversely, metastases from carcinoma NOS featured, for the most part, a necrotic background and solid group architecture; none of the remaining 28 recorded cytomorphological features were described. Therefore, in these patients, a second level of diagnoses was not achieved.

Regarding the role of ancillary techniques, we prepared a much higher percentage of CBs in the naïve than in the oncological group. In fact, the role of ICC staining is more relevant in patients without a medical history of malignancies. Nonetheless, it is interesting to note that, although CB material was available in 44.87% of cases in the naïve group, a second diagnostic level was achieved in 82.49% of cases. These observations confirm that a comprehensive, clinical, and morphological approach allows cytopathologists to accurately diagnose lymph node metastases and may be especially relevant in settings in which the use of ancillary techniques is limited or not available, as is the case in small institutions and developing countries. This evidence is also confirmed by the considerable cytohistological concordance rates.

In conclusion, lymph node FNC is a fundamental tool in the first‐line evaluation of lymph node enlargement. Our results confirm that, in a metastatic setting, FNC can reliably identify not only non‐haematopoietic malignancies, but also the origin of primary tumours. Despite the high number of cases we analysed over a 10‐year period in our referral cytopathologist‐run FNC clinic, this study does have some limitations arising from its retrospective nature and the prevalence of a specific lymph node topography related to the institutional expertise. Nonetheless, our present data should be viewed by cytopathologists as a roadmap for dealing with the diagnostic difficulty posed by the complexity of lymphadenopathies.

## AUTHOR CONTRIBUTIONS

Conceptualisation: G.A., A.I., E.V., and G.T. Resources: G.A. Writing—original draft preparation: E.V., G.A., and G.T. Writing—review and editing: all authors. All authors have read and agreed to the published version of the manuscript.

## CONFLICT OF INTEREST

Giancarlo Troncone received personal fees (as speaker's bureau or advisor) from Roche, MSD, Pfizer, and Bayer, for work unrelated to the current paper. Elena Vigliar received personal fees as advisor from Diaceutics, for work unrelated to the current study. Pasquale Pisapia Troncone received personal fees (as speaker's bureau) from Novartis for work unrelated to the current study. The other authors declare no potential conflicts of interest.

## Data Availability

Data sharing is not applicable to this article as no new data were created or analysed in this study.
